# Risk factors for liver Cancer in HIV endemic areas of Western Kenya

**DOI:** 10.1186/s13027-018-0214-5

**Published:** 2018-12-22

**Authors:** Amos Otedo, Kenneth O. Simbiri, Vincent Were, Omollo Ongati, Benson A. Estambale

**Affiliations:** 1 Jaramogi Oginga Odinga University of Science and Technology, P.O., Box, 210-40601, Bondo,, Kenya; 2 Temple University, Philadelphia, PA USA; 3 Kenya Medical Research Institute, P.O. Box, Kisumu, 1578-40100 Kenya

**Keywords:** Risk factors, HCC, HIV, Western Kenya, Multiple regressions

## Abstract

**Background:**

Hepatocellular carcinoma (HCC) is a malignant cancer of the liver and a leading cause of cancer-related mortality worldwide. Human immunodeficiency virus (HIV) has not been known to have a direct causal relationship with HCC despite independently causing inflammation of the liver. However, little is known on risk factors for HCC patients in an HIV endemic area. The objective of this study was to ascertain the risk factors of HCC and its association with HIV infection.

**Methods:**

This was an un-matched case-control study conducted between June 2015 and June 2016 in Kisumu County Referral Hospital, Kenya. The study enrolled 257 patients with HCC cases and 257 controls**.** A multivariable logistic regression analysis was used to assess the risk factors for HIV and controlling for exploratory variables. Independent t-test was used to compare means. Exposure variable with values < 0.05 were considered to be statistically significant.

**Results:**

HCC cases were more likely to be above 35 years old compared to controls (88.3% vs 23.0%), [aOR =51.6, 95% CI (27.8–95.6), *P* < 0.001)]. HBV infected patients have higher risk of HCC (47.1% vs 30.4%), [aOR = 3.3; 95% CI (1.7–5.0), *P* < 0.0001)]. HIV positive patients were more likely to have HCC than HIV negative patients (33.5% vs 10.9%), [aOR =4.3, 95% CI (2.2–8.4)), *P* < 0.001]. Females have lower risk of HCC than males (32.7 vs 23.7) [aOR = 0.2, 95% CI (0.1–0.4), P < 0.001]. The majority of HCC patients were at the time of diagnosis at stages C (35.1%) and D (48.6%) according to the Barcelona Clinic Liver Clinic (BCLC) criteria.

**Conclusion:**

Liver cancer was more frequent among adults and subjects co-infected with HBV and HIV. Thus, HIV represents an additional risk factor for liver cancer in this Kenyan population. Regular health screening of HIV and HBV infected subjects may significantly improve the early diagnosis and effective therapy of liver cancer.

## Introduction

Hepatocellular carcinoma (HCC) is the most common liver malignancy. It develops from the neoplastic transformation of hepatocytes that represents the second largest organ of the body. It is a primary liver cancer derived from primary liver cells and represents the sixth most common neoplasm and the third leading cause of cancer-related mortality worldwide [1, 2]. In sub-Saharan Africa, it is a public health fixture, which is often ignored, under- diagnosed or diagnosed late. Common risk factors for HCC include viral hepatitis B and C (HBV, HCV), aflatoxins, excess alcohol intake, and tobacco. Other risk factors are iron overload, obesity, diabetes mellitus, genetic predisposition, and α_1_-AT deficiency. However, 18 % of the cases are of unknown causes [3, 4]. Occurrence of HCC has a clear geographical distribution, having the highest incidence in East Asia, sub-Saharan Africa and Melanesia, where about 85% of all cases occur due to the high prevalence of HBV infection. HCV nevertheless, is prevalent in the developed countries [1].

The persistence of the risk factors subsequently leads to development of HCC. HIV mono-infection is known to independently cause acute liver inflammation, a precursor to the development of HCC, but its relevance in liver cancer aetiopathogenesis is still unknown [5]. HIV is prevalent in Western Kenya. HIV/AIDS and cancer are the 3rd and 4th causes of mortality respectively with 11,863 and 9436 cases nationally [6, 7]. In 2013, the highest number of deaths caused by HIV/AIDS and cancer were reported in Nyanza region in Western Kenya [6, 7]. Indeed, in Western Kenya prevalence of HBV is 5–8% [8] and that of HIV ranges from 4.3% in Rift valley to 15.1% in Nyanza. Specifically, the prevalence of HIV is particularly high in counties like Homabay 27%, Siaya 16.2%, Migori 16%, Kisumu 15.3%, and relatively low in Kakamega 4.4%, Nandi 5% and Kericho 4%. The overall prevalence of HIV in Kenya is 6.4% [9, 10].

However, the possible interaction between HIV and other etiological agents in the development of HCC has not been fully elucidated to date.

### Materials and methods

Data used in the study were collected from June 2015 to June 2016. Three hundred and thirty one participants (331) were screened. These were patients with abdominal swelling or liver masses who were referred to the liver clinic (Kisumu County and Referral Hospital) for management as a case of liver mass or swelling either from the medical wards, out patient clinic in the hospital, or other surrounding hospitals. The setting was the liver clinic in Kisumu County and Referral Hospital (the only hospital with a liver clinic in western Kenya). Kisumu County Hospital is the only hospital with a liver clinic and a liver specialist. The HCC cases came from the medical wards, outpatient clinic and lower level health facilities and hospitals lacking the infrastructure to take care of Liver diseases. Fpr cases were patients with abdominal swelling or liver mass at the time of referral and were evaluated to ascertain diagnosis of HCC. Cases and controls were recruited consecutively into the study. Over the one-year study period, the patients were being included into the study as per their HCC status. The controls were also referred to the liver clinic for management of the risk factors of HCC but they did not have HCC. Thus, the controls did not have signs of liver disease, but had risk factors of liver disease.

Each patient signed an informed consent, was clinically examined by the researcher, biodata taken, weight, height, then ultrasound of the abdomen (if they did not have as at referral) was done. Blood was then taken to identify the risk factors of HCC. Liver enzyme levels (alt and ast), HIV (if they did not know their HIV status), inr, alpha-fetoproteins, CD4 + ve cell counts, platelet counts and urine for aflatoxins were analyzed. Liver biopsy was done with normal coagulation profile. Some patients had C-T scan of the abdomen as a diagnostic modality for liver cancer and in such cases, only blood works and urine aflatoxin tests were completed.

The target number was 257 but the total referred to the clinic was 331 within the period the study was carried out. Hence 331 subjects were screened, examined to rule out HCC. Of these, 257 (178 males and 79 females) with HCC were included in the study. Two hundred and fifty seven (257) participants were included as controls. The controls had risk factors for HCC, such as HBV infection and alcohol use but no HCC. They were recruited from blood transfusion services, medical outpatient department, liver clinic and medical wards. A standard questionnaire administered to each study participant included background demographics- bio-data, weight, height, symptoms, signs and duration of HCC as well as relevant family history. All participants were then clinically assessed and an abdominal ultrasound performed to diagnose HCC and to define the stage in accordance to the BCLC criteria [11, 12]. The hall mark of development of HCC is inflammation of the liver, hence, the controls with risk factors were the best group since they had a factor which can cause liver inflammation; even though majority (of the controls) had normal liver enzymes manifesting they were not having liver inflammation at that time of referral. All patient were clinically examined to make certain there was a liver mass. Additionally, ultrasound of the abdomen was used to diagnose HCC and other liver masses which were not HCC. Further, alpha-fetoprotein levels were measured in all patients in the study for those with HCC and controls. HCC was diagnosed by ultrasound of the liver for signs of liver cancer and elevated blood alpha-fetoprotein levels.

Under aseptic technique, a venipuncture was done and blood samples collected for the following investigations: triple serology for HBV, HCV and HIV, for viral markers HBsAg, anti-HCV and anti-HIV, anti-HBc-IgG, complete blood count, CD4+ cell count and ELISA for HIV (for participants with unknown sero-status for HIV), clinical chemistry [alanine and aspartate transaminases (ALT, AST) and random blood sugar (RBS)], alpha-fetoproteins (α-FPs), international normalizing ratio (INR) and urine for aflatoxin assay. Liver biopsy was done in a few patients, 13, who had normal coagulation profile, evidenced by a normal INR and platelet count. Biopsy was done under ultrasound guidance using a wide bore trucut needle and the specimen processed and examined histologically for evidence of liver cancer. Where liver biopsy was not feasible due to coagulation disorders, a presumptive diagnosis of HCC was made depending on both the alpha-fetoprotein levels and features of HCC on liver ultrasound or abdominal C-T scan. A diagnostic testing and counseling was sustained for all participants who tested positive for HIV and all patients with HCC. Family history of liver cancer was taken to evaluate a probable genetic aetiology of HCC.

HIV test was done using the Vidas HIV duo Ultra (HIV 5 series) method while CD4+ cell count was determined using fluorescent activated cell sorter (FACS) flow cytometry method. Complete blood count was done using the Coulter counter machine, liver function tests- ALT and AST were analyzed using the Mindray method (Mindray series analyzer 2013). International normalizing ratio (INR) assay was done using the in-vitro thromboplastin reagent while alpha-fetoprotein assay (α-FP) was done using VIDAS®, AFP, 06991 L system. Anti-HBc IgG was analyzed using VIDAS® Anti-HBc Total II (HBCT) automated qualitative test. Aflatoxin assay was done from urine sample using competitive ELISA techniques. Data analysis was performed using the softwares STATA version 14. Statistical analysis was completed using multiple logistic regression analysis.

## Intervention

Participants were offered appropriate medical care at the liver clinic and medical wards at Kisumu County and Referral Hospital. HCC was appropriately managed conservatively (with low protein, high carbohydrate diet, low sodium diet = 0.06 g/day, controlled fluid intake, spironolactone, simepar, dihydrocodeine (DF118) and lactulose). Highly active anti-retroviral therapy (HAART) was promptly initiated (within 7–29 days, for participants who were HIV positive and HAART naïve) or continued where it had been initiated in patients who had tested positive for HIV.

## Results

**Fig. 1:**
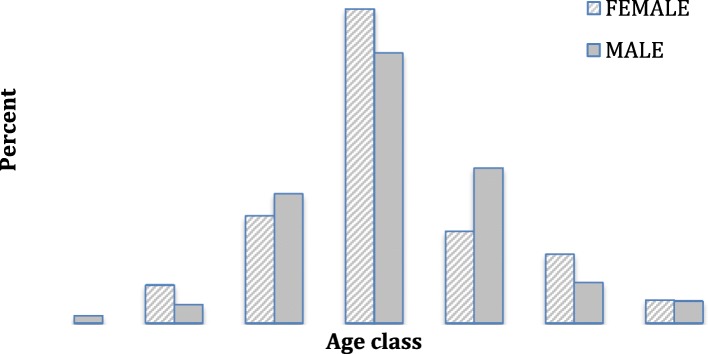
Age distribution of male and female participants with HCC. The figure shows the age and sex distribution of the participants in the study

Two hundred and fifty seven participants (178 males and 79 Female) with HCC and similar number of controls (196 M and 61 F) were recruited into the study. A total of 74 individuals with hemangiomas, liver abscess and adenomas were excluded from the study. The male: female ratio was 2.2:1 for HCC cases and 3.2: 1 for controls. Mean age was 46.2 ± 25.1 years (range 15–80) for cases and 37.4 ± 14.99 years (22–58) for controls. Majority of HCC cases were between the ages 35–60 years (Table 1 and Fig. 1). About 2.7% of HCC subjects had a history of excessive alcohol consumption as compared to 8.2% controls (*p* < 0.617) which is not statistically significant.

**Table 1:** Baseline characteristics: biodata, clinical and laboratory profile of the study patients and controls

Parameter	Cases–N=257 (Range/%)	Controls –N=257 (Range/%)	*P*–value
Age (years) mean ± SD	46.2 ± 25.1 (range 15-80)	37.41 ± 14.9 (22-58)	<0.0001
CD4+ cell (350-1600) /μl	237.7 ± 96.4 [range 50-417]	626.3 ± 102.32 (409-910)	<0.0001
ALT (5-37 IU/L) Mean ±SD	193.5 ± 215.9 [range 2.9-1327]	40.1 ± 2.3 (2-57)	<0.0001
AST (5-40 IU/L) Mean ±SD	173.0 ± 142 [range 14-600]	36.2 ± 7.8 (3-51)	<0.0001
α-FP (0-9ng/ml) Mean ±SD	14,205 ± 58,299[0.5-433,879]	5 ± 2.9 (0-8)	<0.0001
INR (0.67-1.01) Mean ±SD	1.6 ± 0.5 [range 1.0-2.9]	0.9 ± 0.2 (0.5-1.01)	<0.0001
Aflatoxins(1.0-5.0 ppb)	28.35 ppb [2(0.77%)]	0	N/A
BMI (kg/m^2^) Mean ±SD	21.6 ± 3.1 (range 17.64 – 33.87)	20.7 ± 2.3 (59-82)	0.667
HBV mono-infection	40 (15.6%)	179 (69.6%)	<0.0001
HCV mono-infection	1 (0.38%)	0	N/A
HIV mono-infection	51 (19.8%)	28 (10.9%)	0.3097
HBV/HIV co-infection	58 (22.6%)	0	N/A
HCV/HIV co-infection	0	0	N/A
HCC No risk factor or HIV	93 (36.1%)	10 (3.4%)	0.0365
Excess alcohol intake	7 (2.7%)	21(8.2)	0.6173
Positive family history (genetic) of HCC	5 (2%)	0	N/A

The mean CD4+ cell count among HCC cases was low with a mean of 237.7 ± 96.4 [range 50–417] cells/μl while control had 626.3 ± 267.6 (340–910) cells/μl, (*p* < 0.001). Mean ALT and AST were high, 193.5 ± 215.9 [normal range 5–37 IU/L] and 173.0 ± 142 [normal range 5–40 IU/L] for cases and low, 40.1 ± 2.3 IU/L and 36.2 ± 7.8 IU/L for controls respectively, (*p* < 0.001), indicating liver inflammation in the HCC patients. Mean alpha-fetoprotein (α-FP) was high, 14,205 ± 58,299 [normal range of 0-9 ng/ml] compared to 5 ± 2.9 (0–8), (*p* < 0.001), in control. The mean international normalizing ratio (INR) was 1.6 ± 0.8 [normal range 0.67–1.01] in the cases and 0.9 ± 0.2 in the control, (*p* < 0.001). Aflatoxin was found in only 2 (0.77%) of the cases and none of the controls. Body mass index was 21.6 ± 3.1 in cases and 20.7 ± 2.3 in controls and statistically there was no significant difference on BMI between HCC cases and controls, (*p* = 0.667). Mean α–FP was high at 14,205 ± 58,299 [0.5–433,879] in HCC patients and 5 ± 2.9 (0–8) in controls, (p < 0.001). Hepatitis C virus, high body mass index (BMI > 30.0 Kg/m^2^) and genetic factors were each associated with 1 (0.38%), 5(1.9%), and 5(1.9%) respectively. 93 (36.2%) of the study participants had HCC without a known risk factor or HIV.

### Clinical staging and distribution of study

The Barcelona Clinic Liver Clinic (BCLC) criteria as described by Cilo U., et al. and Jordi Bruit, et al., [11, 12] was used for staging HCC. No patient presented in clinical stage 0, 5 patients presented in stage A, 37 patients in stage B, 90 patients in stage C, and 125 patients in stage D. The majority of HCC patients were at stages C (35.1%) and D (48.6%) at the time of diagnosis. Mean duration of abdominal swellings was 3.6 ±  2.66 (range 1–8) months.

### Multivariate logistic regression analysis of risk factors for liver cancer (Table 2)

The potential risk factors in 257 cases of HCC and 257 controls were subjected to multiple logistic regression analysis where having HCC (case) was a dependent variable against the controls. The significant risk factors for HCC compared to controls, were being older than 35 years (88.3% vs 11.7%), [aOR =51.6; 95% confidence interval (CI) (27.8–95.6), (*p* < 0.001)]; to be infected with HBV (47.1% vs 30.4%), [aOR = 3.3; 95% confidence interval (CI) (1.7–5.0), (*p* < 0.0001)], to be infected with HIV (33.5% vs 10.9%), [aOR =4.3, 95% confidence interval (CI) (2.2–8.4)), (*p* < 0.001)]. The risk to develop HCC was higher in males than females (32.7% vs 23.7) [aOR = 0.4, 95% confidence interval (CI) (0.3–0.7), (*p* < 0.001)]. The prevalence of HBV infection was higher in the control because majority of them were referred to the liver clinic with positive HBV status from the blood donor services (101) and the rest 79 were subjects from the wards who had hepatitis due to varied causes which included drugs, herbs, autoimmune state and HIV mono-infection with elevated ALT and AST prior to initiation of HAART. Further stratifications showed that of the 257 cases, 12.8% of them were HIV positive and also had hepatitis compared to 5.5% of controls who were HIV positive and had hepatitis. Cases were 2.6 times more likely to be those who were HIV positive and had hepatitis compared to controls (OR = 2.56; 95% = 1.33–4.90, *p* = 0.005).

**Table 2:** Multivariate logistic regression analysis of risk factors for liver cancer

	Total N=514 (%)	HCC +ve N=257(%)	HCC -ve N=257 (%)	Crude OR (95% CI)	Adjusted OR (95% CI)	*p* value
Age in years						
Youth [<35]	228(44.3)	30(11.7)	198(77.0)	Ref	Ref	
Adult [>35]	286(55.7)	227(88.3)	59(23.0)	37.6(22.6-62.7)	51.6(27.8-95.6)	<0.0001
Sex						
Male	369(71.8)	173(67.3)	196(76.3)	0.4(0.3-0.7)	0.2(0.1-0.4)	<0.0001
Female	145(28.2)	84(32.7)	61(23.7)	Ref	Ref	
Hepatitis B virus infection						
Positive	277(53.9)	98(38.1)	179(69.6)	2.5(1.7-3.3)	3.3(1.67-5.0)	<0.0001
Negative	237(46.1)	159(61.9)	78(30.4)	Ref	Ref	
History of alcohol intake						
Yes	26(5.1)	15(5.9)	21(8.2)	1.3(0.6-2.8)	3.1(0.9-11.1)	0.076
No	488(94.9)	252(98.1)	236(91.8)	Ref	Ref	
HIV infection						
Positive	137(22.2)	109(33.5)	28(10.9)	3.8(2.3-6.0)	4.3(2.2-8.4)	<0.0001
Negative	377(77.8)	148(66.5)	229(89.1)	Ref	Ref	

## Discussion

Hepatocellular carcinoma (HCC) has been identified as the major cause of cancer-related deaths. In addition to the rising incidence rates, and despite advances in diagnosis and treatment in the developed world, the overall prognosis of HCC remains poor with an estimated 5-year survival rate of only 12% [13].

This study shows that the risk factors for HCC in Western Kenya are age > 35 years and to be co-infected with HBV and HIV. This is shown by the statistically significant results in multiple regression analysis of the risk associated with liver cancer (Table 2) showing that HIV positive, HBV positive and age above 35 years are all significantly associated with HCC development. The infectious agents cause acute inflammation of the liver which may determine an increased risk of hepatocyte neoplastic transformation. In fact, persistent liver inflammation may become chronic and evolve into cirrhosis and HCC. It is important to note that 36.0% of the HCC patients and 3.4% of controls in our study had no known risk factors including HIV negativity. On the other hand our results showed that HCC was prevalent in the counties that had high prevalences of HIV infection. Infact 19.5, 16.7, 15.9 and 15.6% of the HCC patients were from Migori, Kisumu, Homabay and Siaya counties, respectively, which are known to have high prevalence of HIV infection. Such observation strongly suggests a casual relationship between HIV infection and HCC development. HIV infection also causes inflammation of the liver [5] as evidenced by elevation of liver transaminases, aspartate and alanine, in the study participants. In the developed world, liver diseases are leading causes of non-AIDS deaths in HIV positive patients [3, 4]. Indeed, in a South African cohort, 4% of HIV positive patients had elevations of liver function tests (liver transaminases, ALT and AST) > 5 times upper limit of normal prior to ARVs [5]**.** HBV/HIV and HCV/HIV co-infections and HCC in HIV positive populations have been demonstrated in the local population [14–17]. Suffice it to note that HIV is not a known risk factor for HCC however HCC has increasingly been identified in the HIV positive populations [3, 14, 15, 18–20].

The high mean ALT and AST shows that there was severe liver inflammation and this predisposes to the development of HCC. While both HBV and HIV independently cause liver inflammation, the co-infection of HBV/HIV can lead to severe liver inflammation which may hasten progression to HCC in the HIV positive populations.

The subjects with HCC who had HIV infection had a low mean CD4+ cell count manifesting severe HIV associated immunosuppression and this may be linked to a higher risk and predictive value of HIV causing HCC. This may be because of the fact that persons co-infected with HIV have faster progression to cirrhosis and decompensated liver disease, especially during immunosuppression and higher viral loads of HBV and/or HCV [21–23].

HIV-induced immune suppression enhances the risk of chronic viral hepatitis; increases viral load of HBV or HCV and may thus hasten the progression to liver cirrhosis and liver cancer. The exact risk of HCC in HIV and HBV/or HCV co-infected patients remains to be fully assessed [21, 24–26]. Moreover, HBV infection is known to persist in 25% of HIV-infected adults compared to < 5% of adults without HIV infection [26]. Like in all cancers, HCC is characterized by a multistage process where an increasing number of mutations accumulate in the genomic DNA causing uncontrolled cell growth. The genetic alterations can be caused by external agents, such as hepatitis viruses, or by excessive cell replication during chronic liver tissue regeneration in chronic hepatitis, which increases the risk of replication errors in the genes [15]. With the prolonged life for HIV/AIDS patients under HAART treatment, it is noted that liver diseases including liver cancers are becoming a common morbidity in the HIV positive population [18, 27–29]. Chronic liver diseases including liver cirrhosis and HCC affect millions of HIV-infected patients who are co-infected with HBV or HCV [5, 21, 29, 30]**.** These end-stage liver diseases account for up to 50% of deaths among people with HIV infection [3–5, 31, 32]. However, there are few data in the developing world on the effects of co-infection with HBV and HIV on risk for HCC [15, 21, 22].

Experiments have demonstrated that there is a possible direct pathway of progression from acute liver inflammation to non-cirrhotic HCC as shown in Figure 3 [33]. The relevance of HIV mono-infection or in interaction with other known risk factors or risk factors in the environment like arsenic, in causing HCC has not been fully elucidated and evaluated and this may predispose to HCC in environments where the risk factors co-exist and interact as demonstrated in this study (Table 2). [33].

Some of the HIV genes enhance production of pro-inflammatory cytokines that sustain chronic inflammation of the liver and subsequently associated with development of HCC and indeed all cancers [34, 35]. The pro-inflammatory cytokine mediators include tumor necrosis factor-alpha (TNF-α), interferon alpha (IFN-α), interleukin-2 and -8 (IL-2 and IL-8), causing inflammation and contribute to cancer development, primarily by causing oxidative stress and DNA damage. Pro-inflammatory cytokines induce the expression of genes involved in cell proliferation, apoptosis, and carcinogenesis causing further production of pro-inflammatory cytokines and worsen the inflammation of the liver [36–38].

The known and unknown risk factors and HIV may contribute to mutations in the genes and result in HCC. In HIV-infected persons, systemic immune activation and CD4+ T-cell function are inextricably linked to immunosenescence which lead to activation-induced cell death [36]. Immunosenescence is important in the pathogenesis of conditions where inflammation represents a significant risk factor, such as atherosclerosis and cardiovascular disease (CVD), neurodegeneration and cancer [39]. Indeed, in the ART era, development of non-AIDS-defining, age-related co-morbidities such as osteoporosis, atherosclerosis, cancers and neurocognitive disease increase is a major cause of morbidity and mortality in HIV-infected persons [36]. The Strategies for Management of Antiretroviral Therapy (SMART) study demonstrated that deaths were mostly due to non-AIDS-defining malignancies (19%) and CVD (13%), while opportunistic diseases only accounted for 8% [36]. The interplay between HIV/HBV or HIV/HCV infections may lead to aggressive liver inflammation and subsequent development of HCC as demonstrated in the multiple regression analysis (Table 2). All the HBV positive subjects in the study were positive for anti-HBc-IgG manifesting chronic infection.

The control subjects were younger than the HCC cases implying that the cases had been exposed longer to the risk factors and HIV (Tables 1, 2 and Fig. 1).

The Barcelona clinic liver clinic (BCLC) staging showed that the majority of HCC cases were diagnosed at stages C (35.1%) and D (48.6%) demonstrating that most patients presented late when the disease is advanced and at a difficult stage to manage causing high mortality**.**

The subjects had a high mean alfa-fetoproteins (α-FP) of 14,205 ± 58,299 ng/ml. 14.4% subjects had normal α-FPs < 9 ng/ml, but had liver ultrasound features suggestive of HCC. High serum levels of alpha-fetoprotein have been found in 60–70% of patients with HCC [40, 41]. For an elevation of alpha-fetoprotein ≥200 and 400 ng/mL the specificity is 100% in both cases, with a sensitivity of 36.3 and 20.2%, respectively [42–44]. Ultrasound imaging is commonly applied in addition to, or in place of, α-FP to help detect small hepatic tumors < 3 cm. While ultrasound was used as a modality for diagnosis of HCC because of availability, C-T scan identifies about 70% of HCC lesions and MRI is the most sensitive and identifies almost 80% of tumors, including 63% of tumors < 2 cm [27]**.**

The widespread use of ultrasound as a surveillance tool relates to its non-invasive nature, high availability and low cost. In combination with α-FP, the positive predictive value (PPV) can be as high as 94% [27, 45, 46]. This exemplifies the importance of combining the two diagnostic modalities in a resource limited setting to make a diagnosis of HCC and more so in HCC subjects with abnormal coagulation profiles where liver biopsy is technically not feasible. When the α-FP blood test strongly indicates HCC and other test results are typical of HCC, a biopsy may not be needed.

Additionally, it should be noted that 40% of HIV positive patients with a nadir CD4+ cell count of less than 200 cells/ml have a potential of developing non-infectious co-morbidities (NICMs) including liver cancer [47]**.** Low CD4+ cell counts predisposes them to develop cancers hence the need for a close surveillance.

**Fig. 2:**
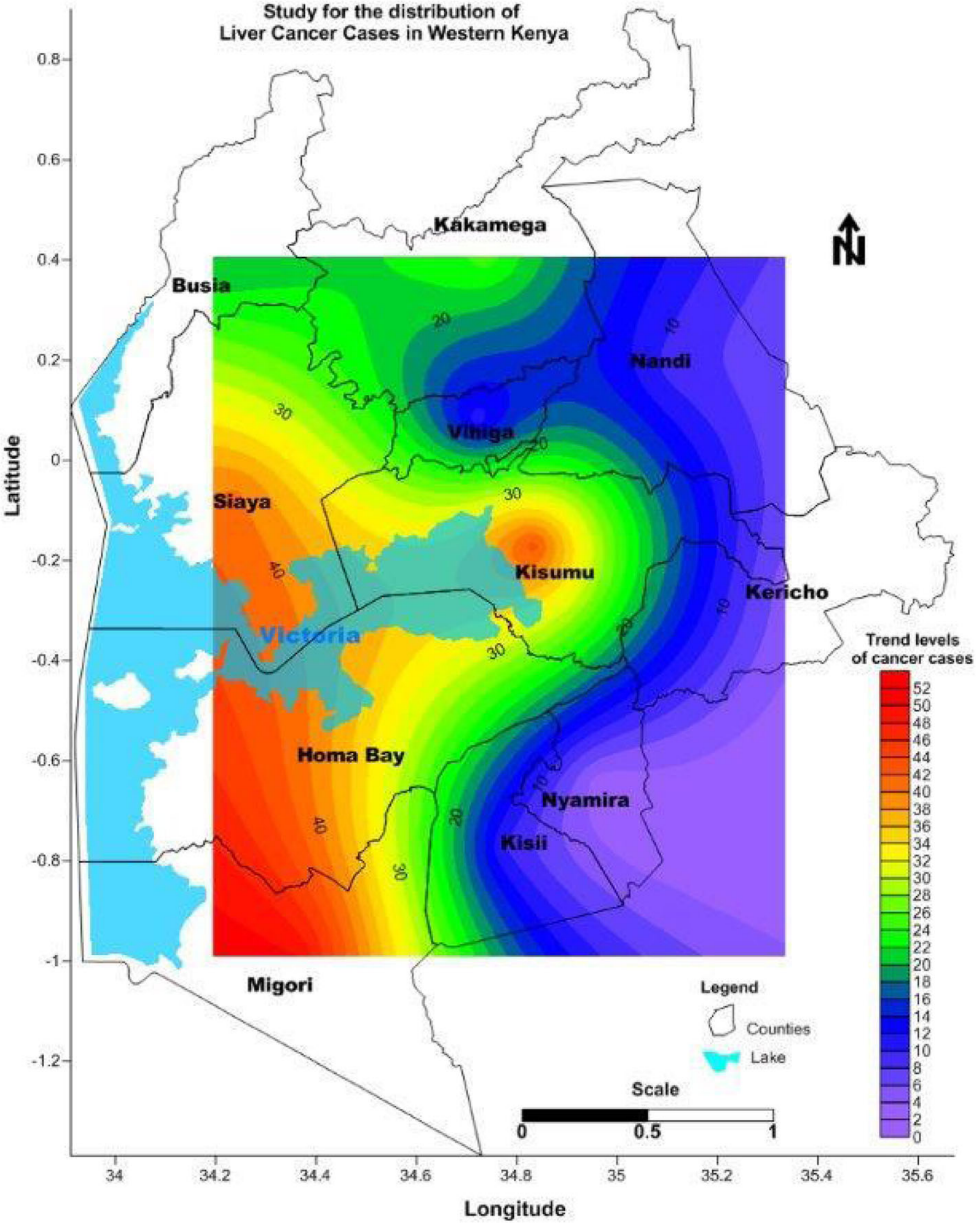
Map of western Kenya showing the trends of HCC. The map shows the distribution of liver cancer cases in western Kenya

The trend of liver cancer in Western Kenya tends to be concentrated along the shores of Lake Victoria as shown in Fig. 2. Since Western Kenya is endemic for HBV at 5–8% [8] and also has counties with high prevalence of HIV including Homabay, Kisumu, Migori and Siaya, this may be attributed to a potential interaction of the risk factors between HIV, HBV and or the heavy metals in water. Globally, there is an increasing trend of primary liver cancer in the developed world due to early exposure to HCV and a concurrent increasing prevalence of obesity and diabetes mellitus during the same time periods. However, HBV and aflatoxins have been noted to be the risk factor in Uganda and the developing world and sub-Saharan Africa (SSA) [48, 49]. There is also a potential increase of HCC after prolonged survival of effectively treated HIV infected persons hence, continued surveillance will be vital to monitor trends [48].

There may be a causal etiology between heavy metals in water and HCC and studies have shown a strong evidence supporting a causal relationship between arsenic in drinking water and both lung and liver cancers [50]. 36.1% of cases of HCC did not have the known risk factors or HIV (Table 1) and these could be due to heavy metals in water. In Migori County, geological study showed that the average concentration of the heavy toxic metals i.e. arsenic, lead, titanium and zinc were above 50 mg/Kg, as recommended by World Health Organization [51]. Further evaluation should therefore be done in these counties to delineate the probable aetiology of HCC and the possible interaction of the multiple risk factors of HCC.

In conclusion, the findings in this study establishes that adults and subjects having HBV and HIV infections are at a higher risk of developing HCC in resource limited setting in Western Kenya.
